# Multifocal Breast Cancer With Discordant Molecular Profiles in a Patient With NF1 and MUTYH Germline Variants: A Case Report

**DOI:** 10.7759/cureus.101562

**Published:** 2026-01-14

**Authors:** Lisa Su, Alan Y Lu, Mohan Narasimhamurthy

**Affiliations:** 1 Pathology and Laboratory Medicine, The University of Tennessee Health Science Center, Memphis, USA; 2 Pathology, Microbiology, and Immunology, Vanderbilt University Medical Center, Nashville, USA

**Keywords:** her2-positive, multifocal breast cancer, mutyh mutation, nf1 mutation, triple-negative breast, tumor heterogeneity, variant of uncertain significance (vus)

## Abstract

The identification of synchronous breast cancers with inter-tumor heterogeneity presents significant diagnostic and therapeutic challenges, particularly in cases of discordant biomarker profiles. We report the case of a 66-year-old African American female with a significant family history of cancer, including two sisters diagnosed with breast cancer at ages 29 and 32. Diagnostic imaging revealed two synchronous right breast masses with similar high-grade histomorphology but discordant molecular profiles, with the larger focus being hormone receptor-negative and human epidermal growth factor receptor 2 (HER2)-positive, and the smaller focus being triple-negative. Initial germline testing identified a heterozygous variant of uncertain significance (VUS) in *NF1* (c.2643G>A, p.Met881Ile). Subsequent testing with an expanded panel identified a pathogenic variant in *MUTYH* (c.1187G>A, p.G396D). The patient was treated with neoadjuvant chemotherapy followed by unilateral mastectomy. This case illustrates the clinical utility of evaluating all foci in multifocal high-grade disease to ensure appropriate systemic therapy and highlights the challenges of interpreting germline variants in the absence of well-described genotype-phenotype associations.

## Introduction

Inter-tumor heterogeneity in multiple synchronous breast cancers presents diagnostic and therapeutic challenges, particularly when discordant biomarker profiles are identified. Specifically, discordance in human epidermal growth factor receptor 2 (HER2) expression between tumor foci can alter neoadjuvant treatment strategies since therapeutic decisions are dependent on the focus with the most aggressive biological behavior. The presence of high-grade multifocal disease, especially in the context of a strong family history, frequently prompts an investigation into an underlying genetic predisposition.

While the majority of breast cancers are sporadic, approximately 5-10% [1] have a hereditary component involving high-penetrance genes such as *BRCA1* and *BRCA2*. The advent of modern next-generation sequencing (NGS)-based panels has expanded the search for susceptibility variants beyond those of high penetrance. While these panels offer high sensitivity, they also frequently identify variants of uncertain significance (VUS) and incidental findings, complicating clinical interpretation and genetic counseling.

Here, we report a case of synchronous multifocal invasive breast carcinomas with discordant molecular profiles (the larger focus being HER2-positive and the smaller focus being triple negative) in a patient with a significant family history of breast cancer and germline findings of an *NF1* VUS and a *MUTYH* pathogenic variant (PV). We discuss the diagnostic and therapeutic considerations for multifocal disease with discordant molecular profiles and provide a review of *NF1* and *MUTYH* variants in the context of breast cancer susceptibility.

## Case presentation

The patient is a 66-year-old African American female with a history of Crohn's disease, status post subtotal colectomy for obstruction, and monoclonal gammopathy of undetermined significance (MGUS). She has a strong family history of breast cancer, including two sisters diagnosed at ages 29 and 32, her mother at age 70, and her paternal grandmother in her 80s. She presented to the breast surgery clinic with a palpable right breast mass that had been present for several months.

**Figure 1:**
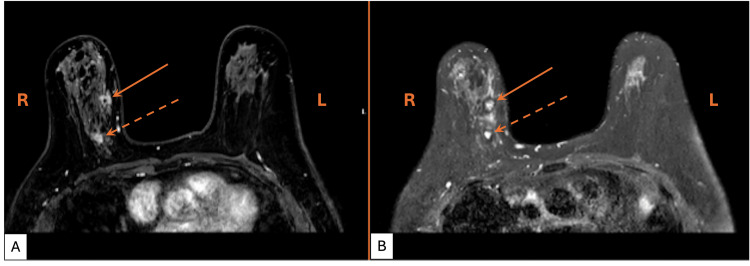
Diagnostic breast MRI showing two suspicious masses A) T1-weighted and B) T2-weighted axial breast magnetic resonance images. R, right side of patient; L, left side of patient. Solid arrow: there is an irregular, heterogeneously enhancing mass with spiculated margins measuring 28 x 19 x 11 mm at the 1 o'clock position in the upper inner quadrant of the right breast, 58 mm from the nipple-areolar complex. Dashed arrow: there is a second mass measuring 11 mm in the longest dimension at the 2 o'clock position, approximately 21 mm posterior and inferior to the index lesion. There is a non-mass enhancement extending between these two lesions spanning a total anterior-posterior extent of 50 mm.

Diagnostic mammography and targeted right breast ultrasound revealed numerous discrete, irregular, and hypoechoic masses with angulated margins and internal vascularity in the right upper quadrant. Subsequent magnetic resonance imaging (MRI) demonstrated two masses within the right breast (Figure 1). Two ultrasound-guided needle core biopsies were performed, targeting the index mass at the 1 o'clock position, 6 cm from the nipple, and the second at the 2 o'clock position, 5 cm from the nipple. 

**Figure 2:**
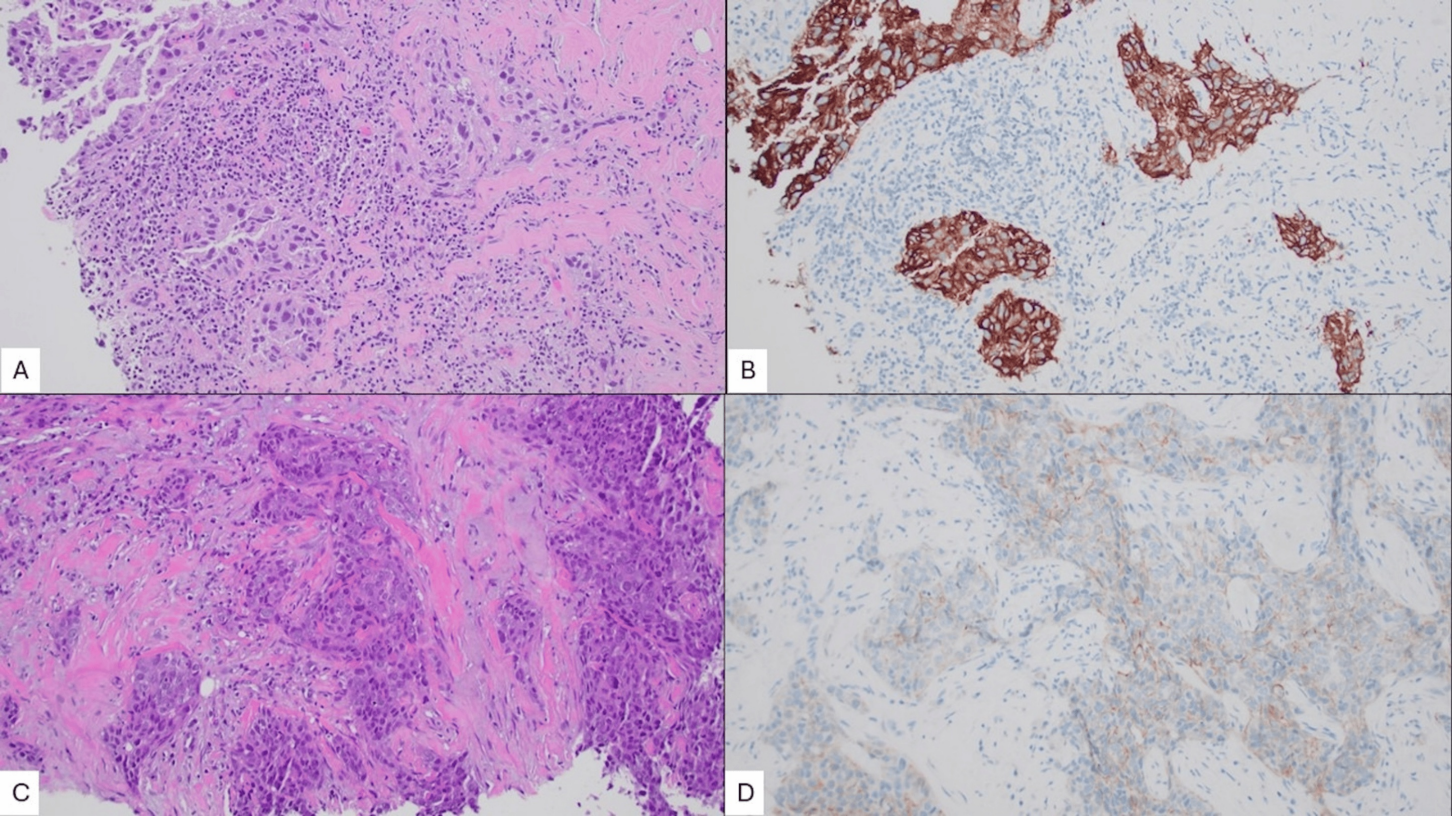
Two foci of invasive breast carcinoma of no special type (IBC-NST) A) Hematoxylin & Eosin (H&E), 200x magnification; the breast mass at the 1 o'clock position, 5 cm from the nipple, shows infiltrative nests of tumor cells with high combined histologic grade, and a dense lymphoplasmacytic stromal infiltrate. B) Human epidermal growth factor receptor 2 (HER2), 200x magnification; the 1 o'clock mass shows a positive HER2 staining pattern (3+) with intense, complete, circumferential membrane staining in essentially 100% of cells. C) H&E, 200x magnification; the breast mass at the 2 o'clock position, 6 cm from the nipple, shows infiltrative nests of tumor cells with high combined histologic grade, and desmoplastic stromal response with no significant immune infiltrate. D) HER2, 200x magnification; the 2 o'clock mass shows a negative HER2 staining pattern (1+) with partial membrane staining in >10% of tumor cells.

Both biopsies revealed infiltrating cohesive nests of high-grade malignant cells with loss of myoepithelial cells, as demonstrated by absent p63 and calponin immunohistochemical staining throughout the invasive component. The tumor cells were strongly positive for cytokeratin 7 (CK7) and SRY-related HMG-box 10 (SOX10), with patchy positivity for GATA-binding protein (GATA-3), supporting the diagnosis of invasive breast carcinoma. The index mass at the 1 o'clock position had a background of high-grade ductal carcinoma in situ (DCIS), as well as a dense stromal lymphocytic infiltrate (Figure 2A). The mass at the 2 o'clock position (Figure 2C) did not show a significant immune response. Both masses were diagnosed as invasive breast carcinoma of no special type (IBC-NST), with a high combined histologic grade in each case.

Immunohistochemical studies for receptor status revealed that both masses were negative for estrogen (ER) and progesterone receptor (PR). The index mass at 1 o'clock showed HER2 overexpression (Figure 2B), whereas the 2 o'clock mass showed a negative expression pattern (Figure 2D). Subsequent dual-probe fluorescence in situ hybridization (FISH) studies of the 1 o'clock mass confirmed HER2 amplification, with a HER2 to centromere enumeration probe 17 (CEP17) ratio of 9.8 and an average *ERBB2* copy number of 24.5 signals per cell. These results meet the criteria for HER2 positivity (HER2/CEP17 ratio ≥2.0 with an *ERBB2* copy number ≥4.0 signals/cell) per American Society of Clinical Oncology (ASCO) and College of American Pathologists (CAP) guidelines [2].

**Figure 3:**
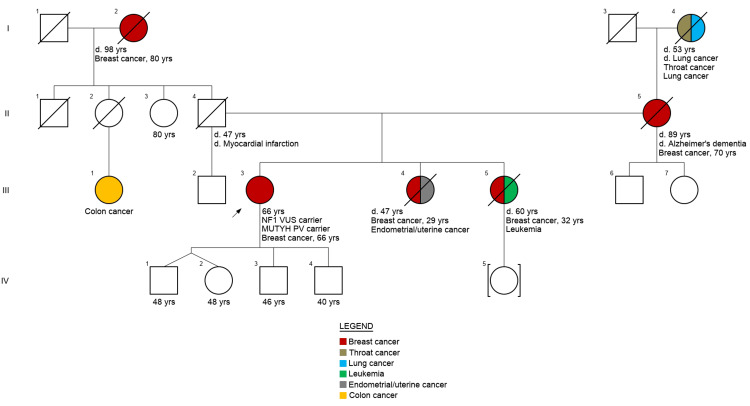
Pedigree illustrating the patient's multi-generational history of cancers The proband (III-3) is marked with an arrow with descriptors underneath the symbol that the patient was diagnosed with multifocal breast cancer at age 66 and is a carrier of an *NF1* VUS and a *MUTYH* PV. Notably, the pedigree describes early-onset breast cancer in two sisters (diagnosed at 29 and 32 years) who are both deceased from other reasons; maternal breast cancer at age 70, who is deceased from Alzheimer's dementia; and paternal grandmother breast cancer at age 80, who is deceased from other reasons. Additional malignancies in the family include endometrial cancer in one of the patient's sisters due to tamoxifen therapy for breast cancer, leukemia in the patient's other sister, colon cancer in a paternal cousin, and lung and throat cancer in her maternal grandmother. d. - age/cause of death (if known); VUS - variant of uncertain significance; PV - pathogenic variant Created with Invitae's Family History Tool [4]

Given the patient's multifocal disease, aggressive biomarker profiles, and family history of early-onset breast cancer (see pedigree in Figure 3), she met National Comprehensive Cancer Network (NCCN) guidelines for hereditary cancer genetic testing [3]. The patient underwent initial testing with a 13-gene targeted panel (BRCAplus, Ambry Genetics) that targets a subset of the 21 NCCN-recognized genes [3] for which established risk-reduction, surveillance, and other clinical management strategies exist for breast, ovarian, and/or pancreatic cancers. Initial testing identified a heterozygous variant of unknown significance (VUS) in *NF1* (c.2643G>A, p.Met881Ile). Subsequent testing with a 76-gene expanded panel (CancerNext-Expanded, Ambry Genetics) identified a heterozygous pathogenic variant in *MUTYH* (c.1187G>A, p.G396D) in addition to the previously noted *NF1* VUS. The full list of genes included on both panels is listed in the Appendix. 

Despite the patient's family history of early-onset breast cancer, there is no record or knowledge of genetic testing for other affected family members. The patient's family members were informed of her *MUTYH* carrier status. The patient's genetic results did not alter clinical management, however, as current evidence does not support a definitive association between monoallelic *MUTYH* pathogenic variants and increased breast cancer risk [5]. The patient was treated with neoadjuvant chemotherapy followed by unilateral mastectomy and is currently doing well on follow-up.

## Discussion

The reported prevalence of HER2 discordance among synchronous breast cancer foci varies widely, with studies reporting rates between 0% [6] and 16.1% [7]. Tumor heterogeneity fuels ongoing debate regarding whether to perform biomarker testing on all foci or only the largest focus in multifocal and multicentric disease. Current CAP guidelines state that biomarker testing may be limited to the largest focus, particularly when morphology and grade among foci are concordant [8], and the available data indicate that the largest tumor typically harbors the most clinically meaningful information, such as HER2 overexpression (>95% of cases) [7, 9-10]. Interestingly, in our patient's case, the smaller of the two foci was triple-negative, prompting initiation of neoadjuvant chemotherapy. This finding highlights the utility of multifocal sampling and biomarker testing, especially when the foci have high-grade histomorphology, to ensure that systemic treatment is tailored to the most aggressive molecular subtype.

This case further illustrates the challenges of interpreting germline variants of uncertain clinical utility in the setting of a suggestive family history. Despite a clinical suspicion of hereditary cancer due to the patient's two sisters with early-onset disease, expanded testing identified only an *NF1* VUS and a monoallelic *MUTYH* PV, neither of which is known to be a high-penetrance breast cancer susceptibility gene. *NF1* is a tumor suppressor gene that encodes the protein neurofibromin, a negative regulator of the Ras signaling pathway. Pathogenic *NF1* variants result in neurofibromatosis type 1, an autosomal dominant multisystem tumor-predisposition syndrome characterized by diagnostic clinical features such as café-au-lait macules, axillary or inguinal freckling, neurofibromas, optic pathway glioma, iris Lisch nodules, and specific osseous lesions [11]. The role of *NF1* variants in breast cancer susceptibility is being investigated since studies have shown that women with neurofibromatosis type 1 have a five-fold increased risk of developing breast cancer before the age of 50 [12] with higher rates of HER2+ cancers and mortality [13]. A large clinical cohort study of over 165,000 patients referred for germline testing with multigene panels from Ambry Genetics suggests that *NF1* PVs, while rare, behave as a moderate-risk breast cancer susceptibility gene [14]. Accordingly, NCCN guidelines recommend that individuals with *NF1* PVs receive annual screening mammograms starting at age 30, with the consideration of adding annual breast MRI with and without contrast from ages 30 to 50 [3].

Translating these established risks to our patient's case remains difficult since she presented with neither a clinical diagnosis of neurofibromatosis type 1 nor a definitive genetic result. The identified *NF1* missense variant (c.2643G>A, p.Met881Ile) is rare (<0.001%) in the general population, based on the Genome Aggregation Database v4.1.0, though notably more prevalent in individuals of African/African American ancestry (0.004%) [15]. Multiple *in silico* prediction tools and available ClinVar submissions [16] support a benign or uncertain clinical significance of this variant (results summarized in Appendix). Furthermore, the patient's age and history of MGUS warrant consideration of clonal hematopoiesis of indeterminate potential (CHIP), which is the expansion of somatic mutations in hematopoietic stem cells that can be incidentally detected on NGS testing and misidentified as germline variants. Ancillary studies, such as tumor sequencing and testing benign, non-blood tissue, would be helpful to differentiate a true germline variant from CHIP and are critical for accurate genetic counseling.

The identified *MUTYH* variant (c.1187G>A, p.G396D; isoform p.G382D) is a well-characterized missense mutation resulting in defective base excision repair. Biallelic *MUTYH* mutations lead to MUTYH-associated polyposis (MAP), an autosomal recessive syndrome characterized by multiple adenomatous colorectal polyps [17-18], and *MUTYH* mutations have been found to be linked to an increased risk of colorectal cancer [19-20]. The risk of extracolonic malignancies such as breast cancer associated with monoallelic *MUTYH* mutations remains debated, however. While such an association was first noted in a Dutch cohort in 2005 [21], the majority of subsequent studies have failed to establish a definitive link [22-27]. Monoallelic *MUTYH* PVs are found at a higher frequency than *NF1* mutants in breast cancer cohorts, but this is attributed to the higher frequency of common *MUTYH* mutations, p.G382D and p.Y179C, in the general population rather than a specific association with breast cancer [14]. As such, current NCCN guidelines do not recommend altered breast screening for *MUTYH* carriers [3].

Although *NF1* and *MUTYH* genes are included on commercial panels, large-scale association studies from global and U.S. populations conducted via the Breast Cancer Association Consortium (BCAC) [28] and the CARRIERS consortium [29] indicate that neither gene is a primary driver of breast cancer. Furthermore, while protein-truncating *NF1* variants are associated with a modest, non-statistically significant increased risk of breast cancer, rare *NF1* missense variants, like the one in our patient, did not result in similar findings [28]. These studies support a core clinical panel of 10 genes (*ATM, BARD1, BRCA1, BRCA2, BRIP1, CHEK2, PALB2, RAD51C, RAD51D,* *TP53*) for testing in patients with breast or ovarian cancer [30].

While the identified *NF1* and *MUTYH* variants offer mechanistic insights, the lack of tumor sequencing and family co-segregation data in this case limits our ability to suggest a genotype-phenotype link. Furthermore, the failure to identify a high-penetrance mutation suggests that the patient's familial clustering of breast cancer is more likely multifactorial in origin rather than due to a monogenic driver. Polygenic risk scores (PRS) offer a promising framework to aggregate small-effect variants into a clinically meaningful measure of genetic susceptibility, and large-scale association studies have successfully utilized PRS to refine risk stratification in *BRCA1* and *BRCA2* PV carriers [31-32]. Future research in diverse, non-European ancestries is essential to ensure these risk-stratification tools are both accurate and equitable.

## Conclusions

In summary, we report a rare case of synchronous HER2-positive and triple-negative breast carcinomas in a patient with a significant family history and interesting germline findings of an *NF1* VUS and monoallelic *MUTYH* PV. This case demonstrates that biomarker discordance in multifocal disease can fundamentally alter systemic therapy, necessitating a low threshold for multifocal sampling and biomarker testing. Furthermore, the case highlights the interpretative challenges of multigene panels, where rare variants and incidental findings can complicate counseling without explaining the underlying family history. We advocate for comprehensive documentation of similar cases alongside large-scale cohort studies and functional assays to refine variant interpretation.

## Appendices

**Table 1:** Comprehensive list of genes evaluated via NGS *Discussed in NCCN guidelines [3] for which established risk-reduction, surveillance, and other clinical management strategies exist for breast, ovarian, and/or pancreatic cancers.

Gene Panel	Genes Assessed
BRCAplus, Ambry Genetics	ATM*, BARD1*, BRCA1*, BRCA2*, CDH1*, CHEK2*, NF1*, PALB2*, PTEN*, RAD51C*, RAD51D*, STK11*, TP53*
CancerNext-Expanded, Ambry Genetics	AIP, ALK, APC, ATM*, AXIN2, BAP1, BARD1*, BMPR1A, BRCA1*, BRCA2*, BRIP1*, CDC73, CDH1*, CDK4, CDKN1B, CDKN2A*, CEBPA, CHEK*2, CTNNA1, DDX41, DICER1, EGFR, EPCAM*, ETV6, FH, FLCN, GATA2, GREM1, HOXB13*, KIT, LZTR1, MAX, MBD4, MITF, MEN1, MET, MLH1*, MSH2*, MSH3, MSH6*, MUTYH, NF1*, NF2, NTHL1, PALB2*, PDGFRA, PHOX2B, PMS2, POLD1, POLE, POT1, PRKAR1A, PTCH1, PTEN*, RAD51C*, RAD51D*, RB1, RET, RUNX1, SDHA, SDHAF2, SDHB, SDHC, SDHD, SMAD4, SMARCA4, SMARCB1, SMARCE1, STK11*, SUFU, TMEM127, TP53*, TSC1, TSC2, VHL, WT1

**Table 2:** Summary of NF1 VUS Abbreviations: HGVS, Human Genome Variation Society; G, guanine; A, alanine; GRCh38, Genome Reference Consortium Human Build 38; Met, methionine; Ile, isoleucine; NCBI, National Center for Biotechnology Information; GRD, GTP-activating protein-related domain; gnomAD, Genome Aggregation Database; REVEL, Rare Exome Variant Ensemble Learner; CADD, Combined Annotation Dependent Depletion; SIFT, Sorting Intolerant From Tolerant; PolyPhen-2, Polymorphism Phenotyping v2

Gene	NF1
DNA change (HGVS) [15]	c.2643G>A (guanine to alanine non-synonymous single-nucleotide substitution at the 2643^rd^ position on the complementary DNA strand)
Variant location (GRCh38) [15]	Chr17: 31229258
Amino acid change (HGVS) [15]	p.Met881Ile (methionine to isoleucine amino acid substitution at the 881^st^ position)
Location in protein (NCBI Reference Sequence: NM_001042492.3) [32]	Outside known functional domains (e.g., not in GRD)
ClinVar classification [15]	Conflicting classifications of pathogenicity: Uncertain significance (4); Benign (1)
Population frequency (gnomAD v4.1.0) [14]	<0.001% (4/1613066) total alleles studied; 0.004% (3/74906) African/African American alleles
REVEL score [15]	0.263 (Indeterminate)
CADD score [15]	19.3 (Borderline-low)
SIFT prediction [15]	Tolerated
PolyPhen-2 score [15]	0.00 (Benign)
